# A Robust Ensemble of Convolutional Neural Networks for the Detection of Monkeypox Disease from Skin Images

**DOI:** 10.3390/s23167134

**Published:** 2023-08-12

**Authors:** Luis Muñoz-Saavedra, Elena Escobar-Linero, Javier Civit-Masot, Francisco Luna-Perejón, Antón Civit, Manuel Domínguez-Morales

**Affiliations:** 1 Architecture and Computer Technology Department (ATC), E.T.S. Ingeniería Informática, Universidad de Sevilla, 41012 Seville, Spain; lmsaavedra@us.es (L.M.-S.); eescobar@us.es (E.E.-L.); mjavier@us.es (J.C.-M.); fluna1@us.es (F.L.-P.); civit@us.es (A.C.); 2 Robotics and Technology of Computers Lab (RTC), E.T.S. Ingeniería Informática, Universidad de Sevilla, 41012 Seville, Spain

**Keywords:** monkeypox, skin disease, database, convolutional neural network, ensemble

## Abstract

Monkeypox is a smallpox-like disease that was declared a global health emergency in July 2022. Because of this resemblance, it is not easy to distinguish a monkeypox rash from other similar diseases; however, due to the novelty of this disease, there are no widely used databases for this purpose with which to develop image-based classification algorithms. Therefore, three significant contributions are proposed in this work: first, the development of a publicly available dataset of monkeypox images; second, the development of a classification system based on convolutional neural networks in order to automatically distinguish monkeypox marks from those produced by other diseases; and, finally, the use of explainable AI tools for ensemble networks. For point 1, free images of monkeypox cases and other diseases have been searched in government databases and processed until we are left with only a section of the skin of the patients in each case. For point 2, various pre-trained models were used as classifiers and, in the second instance, combinations of these were used to form ensembles. And, for point 3, this is the first documented time that an explainable AI technique (like GradCAM) is applied to the results of ensemble networks. Among all the tests, the accuracy reaches 93% in the case of single pre-trained networks, and up to 98% using an ensemble of three networks (ResNet50, EfficientNetB0, and MobileNetV2). Comparing these results with previous work, a substantial improvement in classification accuracy is observed.

## 1. Introduction

The first recorded case of monkeypox dates back to 1958, when two outbreaks of a smallpox-like disease occurred in monkey colonies under investigation. Despite being named “monkeypox”, the origin of the disease remains unknown. However, African rodents and non-human primates (such as monkeys) could harbor the virus and infect humans [1]; and the first human case of monkeypox was reported in 1970. Before the 2022 outbreak, monkeypox cases had been reported in people in several countries in Central and West Africa (almost all cases outside Africa were associated with international travels or animal commerce [2]). According to the last report by the Centre for Disease Control and Prevention (CDC) on 28 July 2022, more than 23,000 cases have been reported worldwide since the beginning of the 2022 outbreak [3]. Regarding those cases, the two countries with the most reported cases are the United States with more than 5800, and Spain with more than 4200. Furthermore, in Spain and Brazil, monkeypox deaths have been reported [4].

Although the spread of this virus does not resemble other recent pandemics such as COVID-19, its expansion in Western countries has been surprising; so, it is important to facilitate a rapid diagnosis of the disease that does not require expensive diagnostic equipment or a long response time. For that purpose, it is necessary that the information it receives can be provided by a non-sanitary user; therefore, the easiest input for a common user is an image, and, in order to be easily captured for an average user, it should be a superficial image of the skin. Furthermore, this type of system must take into account other skin damage caused by similar viruses or other diseases such as smallpox, chickenpox, or measles. However, currently, there is no dataset of close-up skin images, although some datasets of full-body images are available. Therefore, for such large-scale work, it would be necessary to collect an appropriate dataset to design the classifier.

Regarding the development of classifying models, the use of artificial intelligence techniques has been extended in recent years within the field of health: from the analysis of physiological signals [5,6] to the study of bad habits and abnormalities during daily-life activities [7,8]. Furthermore, in relation to this work, the use of techniques derived from AI and machine learning (ML) has wide use in the design of automatic classifiers with medical images, making use of advanced Deep Learning (DL) techniques in CNN. Thus, these techniques have been applied in multiple research works in recent years, obtaining very positive results with a correct diagnosis rate higher than 80% [9,10,11,12], even reaching, in multiple cases, values higher than 95% accuracy [13,14,15,16,17].

However, these classifier systems are tested with a subset of samples and, in future classifications, there are possibilities to obtain the wrong classifications due to samples from other medical centers, or because of using other digitizing devices (among other possible cases) [18]. Moreover, when an AI system is trained, the weights of the neural network connections are very difficult to be understood and do not provide useful information: that is why these systems are referred to as “black boxes” [19].

Because of that, in recent years, the use of Explainable Artificial Intelligent (xAI) technologies has become widespread. These technologies provide information about the objective classification criteria used in the automatic system [20,21]. This is of great importance, not only to detect errors, but also to understand the decisions made. That is why this type of analysis is essential in diagnostic aid systems [16,22,23].

Following the identification of research gaps, the main objectives of this work are described next:

- Designing a dataset of superficial skin photographs containing images of monkeypox cases, healthy people, and people with other types of diseases that produce skin rashes. This dataset is provided publicly to the community.
- Studying various alternatives of classifiers based on convolutional neural networks to distinguish between the three classes described above. These classifiers will be performed by applying transfer learning techniques to pre-trained models.
- Combining different classifier models to form ensemble systems that perform the same task, comparing the results with those obtained previously.
- Evaluating the results obtained by the classifier using xAI techniques. As far as we know, this is the first time it is applied to an ensemble classifier.

The rest of the manuscript is structured as follows: first, a presentation of the latest similar works published is presented; next, the methods used to develop and test the diagnosis-aid system are presented in Section 3, including the description of the dataset developed for this work. The accuracy results obtained after testing the classifier are detailed and discussed in Section 4, with the application of the explainable AI techniques and the comparison with previous works. Finally, in Section 5, the final conclusions of this work and future research lines.

## 2. Background

A global search is performed on the main search engines (Scopus, IEEExplorer, and Google Scholar) with the following search phrase: “monkeypox” AND (“deep learning” OR “convolutional neural network”). Due to the current spread of the monkeypox outbreak, the results have not been filtered or restricted by year. Moreover, those preprints or arXiv/bioRxiv works that are waiting for acceptance are selected as well.

However, the results after the search process and the elimination of articles not focused on the design of a classifier, reflect very few works in this area (focused on only 2022). So, the works used for the comparison will only be those detailed next:

- Ali et al. [24]: In this work, a binary classification of monkeypox and other skin diseases is performed using skin images taken by users. The authors tested several convolutional neural network models such as VGG-16, ResNet50, and Inception-V3. The dataset uses 102 monkeypox images and 126 images of other skin diseases, but it does not include images of healthy skin tissue. The best classifier has an accuracy greater than 82%.
- Ahsan et al. [25]: this work uses a custom dataset formed by the four classes “healthy”, “measles”, “chickenpox” and “monkeypox”, containing 54, 17, 47, and 43 images for each class, respectively. Although the authors use a data augmentation process, the dataset has very few images for some classes, and it is quite unbalanced. However, the developed classifiers are trained only for two classes (“monkeypox” versus “others”, and “monkeypox” versus “chickenpox”). Using a VGG-16 CNN for each implemented system, the authors obtain an 83% accuracy for the “monkeypox” vs. “others” study for the training subset, and a 78% accuracy for the second experiment using the training subset.

We believe that the lack of work in this area is due, first, to the novelty of the global spread of the virus; but, second, it is also due to the lack of a sufficiently balanced and formal public dataset. This latter point is founded on the fact that the works detailed in this section are the same as those named in Section 3.1, when exposing the existing datasets (i.e., each work uses its own dataset). This aspect is one of the main objectives of this work when collecting a new public dataset.

## 3. Materials and Methods

**Figure 1:**
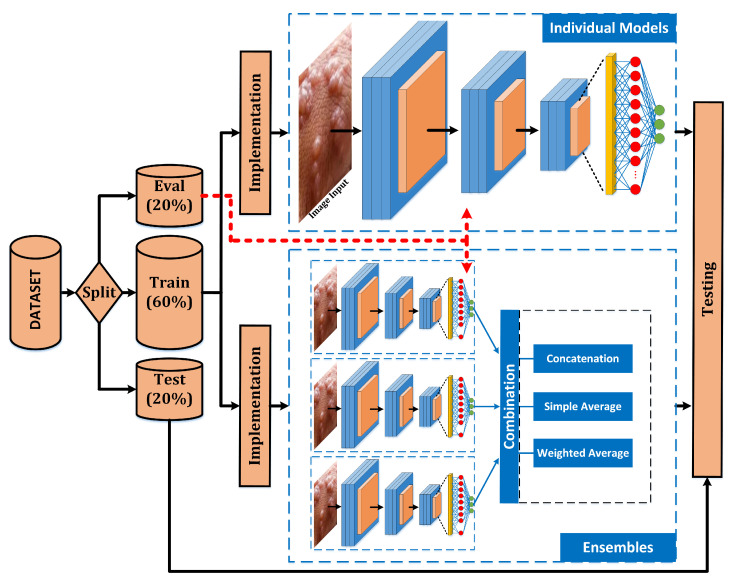
Full system scheme. The main difference between both implementations is: (bottom) each model is evaluated independently using only its own results; and (down) some combinations of three individual models (ensembles) are evaluated by the combination of the individual models.

This section presents the custom dataset used to train and test the classification system, the developed classifiers, and the metrics used to evaluate them. For this purpose, the methodology applied to achieve these goals is summarized in Figure 1. The collected dataset will be used to evaluate some pre-trained models (explained in the next subsections), applying a grid search with several hyperparameter combinations. Moreover, some ensemble models are evaluated too (the selection of these ensembles is detailed in the next subsections). After these testing, the best model will be compared with the previous works.

### 3.1. Dataset

The custom dataset consists of 224 × 244-pixel images in RGB format. Images were obtained from contrasted online media (such as CDC or WHO) and from public datasets. The only public datasets found are the one collected by [24] and the one collected by [25,26]. For the dataset collected in this work, three main premises are taken into account:

- Classes: the dataset needs to distinguish between healthy and ill tissue (with Monkeypox). In addition, it is essential to include other skin diseases to determine the degree of importance of the injury.
- Number of images: It is important to develop a balanced dataset with the same number of images for each class.
- Type of images: To develop a classifier that can be integrated into a mobile device, images with similar characteristics for all classes and highlighting skin lesions (avoiding images taken from a distance or of whole body parts) are required.

Following these premises, the final dataset collected is composed of three classes: “monkeypox”, “healthy”, and “other diseases”. Each class contains 100 images of the skin surface. The summary of the collected dataset can be seen in Table 1, which also contains the comparison with the public dataset found. The dataset collected for this work is publicly accessible [27].

The main problems observed in Table 1 for the previous public datasets are described next:

- Ali et al. [24]: the main problem of this dataset is the image classes. It has only two classes, and, moreover, there is no “Healthy” class, and this absence can lead to failures when evaluating the classifier with healthy tissue images; since there is no class containing it, any image that it classifies is labeled as damaged tissue (by Monkeypox or some other disease). This problem was previously addressed in other works [28].
- Ahsan et al. [25]: this second dataset contains a good number of classes (including “Healthy” ones), but it has two main drawbacks. The first is the total number of images, only surpassing 50 images for the “Healthy” class (54 images) and giving only 17 images for one of the classes (Measles). The second drawback is data balance: the worst case is observed with the “Healthy” class, which has more than three times the images given by the “Measles” class.

Also, for both datasets, the images used do not follow a similarity pattern, including full-body images or images of single body parts (even with images in which more than one person appears). For correct training of the classifier, it would be necessary to discard a significant part of the images in these datasets due to deficiencies such as those indicated, or even cases where two different images overlap.

**Figure 2:**
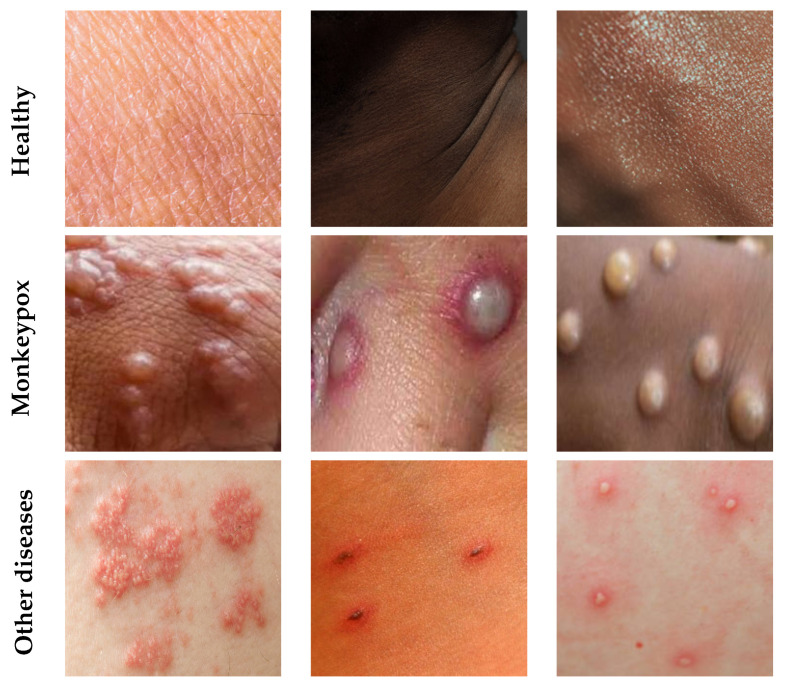
Sample images from MonkeySkin dataset.

Trying to solve these drawbacks, and shown in Table 1, the dataset collected for this work has a perfect balance between classes, which facilitates the classifier training task. In addition, the use of close images of the skin surface for all classes helps to present a more uniform dataset that better represents the information that can be obtained from a photograph with a mobile device. Figure 2 shows some examples of images from the collected dataset.

The images of the dataset are used to train the classifiers, using 60% of the images for training, 20% for validation, and the last 20% for testing. In addition, to improve the training, a data augmentation process is performed only on the images of the training set (performing random rotations of the images until a total of 9000 images are obtained). This division is presented in Table 2.

The justification for using a dataset from online sources is due to the need to have the class of each image already labeled by contrasted sources. However, the objective of this work is to design a classifier that is able to work with images obtained by users using easily accessible vision sensors, such as a cell phone camera. This would provide a quick first response to the user without the need to go to a hospital, performing an initial screening that would reduce the burden on healthcare professionals.

Of course, this preliminary diagnosis should be corroborated by a professional at a later stage and, for this purpose, in the last part of the paper, explainable AI techniques will be presented in order to provide more information to the healthcare professional verifying the classification initially obtained by the diagnostic aid system.

### 3.2. Classifiers

The classifiers evaluated are pre-trained models on which the weights of the first layers of neurons will be maintained and trained on the weights of the last layers (applying transfer learning techniques for this purpose). For them, a preliminary study was carried out by means of a grid search with multiple combinations of custom classifiers. However, two main problems related to it were found:

- The results show that the system required complex architectures to converge, in addition to a very long training process. In those previous tests, an architecture of 10 convolutional layers and 3 dense layers required a total of 100K epochs to obtain an accuracy of around 80% (after ten days). Because of this, we estimate that this type of architecture required more complexity and more training time to obtain acceptable results (at least 95%); in fact, observing the training tendency, obtaining an 85% accuracy would require more than two months (needing more than a year to reach 90%).
- Searching for previous works, it was observed that the trained systems were based on pre-trained models. Therefore, if we wanted to compare ourselves with those works, we had to work with similar systems that would allow us to measure the improvement obtained.

Therefore, because of that, in this work several pre-trained models will be used (applying transfer learning techniques): each model will be independently evaluated, and subsequently combinations of models will be performed to evaluate whether there is an improvement in classification. As for the hyperparameters used, they will be those commonly used for these models, which are a batch size of 32 and a learning rate of 1 × 10${}^{-4}$, with training of 50 epochs. The individual models used, and the ensembles will be presented below.

#### 3.2.1. Individual Models

The individual models used in this work are as follows:

- VGG-16: is a convolutional neural network proposed by [29] of Oxford University, and gained notoriety by winning the ImageNet Large-Scale Visual Recognition Challenge (ILSVRC) in 2014. It is composed of 13 convolutional layers, 5 polling layers, and 3 dense layers.
- VGG-19: It is a variant with more computational layers than VGG-16, therefore, heavier in memory storage and computational requirements. In this case, the number of polling and dense layers are the same, but the convolutional layers increase to 16.
- ResNet50: It was introduced by Microsoft and won the ILSVRC (ImageNet Large-Scale Visual Recognition Challenge) competition in 2015 [30]. Its operation is based on increasing the number of layers by introducing a residual connection, moving on to the next one directly, and improving the learning process. This model is much more complex than the previous one: it has almost 50 convolutional layers, 2 polling layers, and one dense layer.
- MobileNet-V2: is a convolutional neural network architecture that seeks to perform well on mobile devices. It is based on an inverted residual structure in which the residual connections are between the bottleneck layers [31]. In this case, the number of convolution layers is similar to that used by ResNet50 (around 50), as well as the polling layers (1) and the dense layers (1).
- EfficientNet-B0: It is a convolutional neural network architecture and scaling method that uniformly scales all dimensions of depth/width/resolution using a compound coefficient [32]. The base EfficientNet-B0 network is based on the inverted bottleneck residual blocks of MobileNetV2, in addition to squeeze-and-excitation blocks. Finally, in this case, the number of convolutional layers is reduced to 33, increasing the number of polling layers to 17, and the number of dense layers to 33.

#### 3.2.2. Ensemble Classifiers

Therefore, if we design ensembles of 3 nets, we will have a total of 24 possible combinations without repetition. In addition, if we include the three usual types of combination of information (concatenation, simple average, and weighted average, which will be detailed below) we would have a total of 72 possibilities.

Although results could be presented for all of them, we believe that many of these combinations do not contribute anything interesting to this work due to the low classification results.

This is why preliminary tests were carried out and, in this paper, only the three ensembles (with their three information combination mechanisms, for a total of 9 possibilities) with the best classification results are presented.

These three ensembles are listed below, and their results are presented in Section 4.1.2:

- Ensemble 1: VGG-16 + VGG-19 + ResNet50
- Ensemble 2: VGG-16 + ResNet50 + EfficientNet-B0
- Ensemble 3: ResNet50 + EfficientNet-B0 + MobileNet-V2

The models of each ensemble are run in parallel, and the results of each ensemble are subsequently merged. The combinations of these models are performed following the three classical approaches of ensemble neural networks:

- Concatenation Ensemble: This is the most common technique to merge different data sources. A concatenation ensemble receives different inputs, whatever their dimensions, and concatenates them on a given axis. This operation can be dispersive, not allowing the final part of the network to learn important information, or resulting in overfitting.
- Average Ensemble: this can be considered to be the opposite of the concatenation operation. The pooled outputs of the networks are passed through dense layers with a fixed number of neurons to equalize them. In this way, the average is computed. The drawback of this approach is the loss of information caused by the nature of the average operation.
- Weighted Ensemble: This is a special form of average operation, where the tensor outputs are multiplied by a weight and then linearly combined. These weights determine the contribution of each model in the final result, but they are not fixed because these values are optimized during the training process. For the weighted ensemble, we have proceeded to create a function that calculates this weighting in real time. In this case, it creates an average with adaptive weights between the outputs and trains the weights by backtracking just like those of any other layer. Finally, it adjusts them with softmax so that they always add up to 1. This mechanism has been used in previous works such as the one recently published in [33].

### 3.3. Evaluation Metrics

To evaluate the effectiveness of the classification systems, it is common to use different and well-known metrics: accuracy (most-used metric), sensitivity (also known as recall), specificity, precision, and F1${}_{score}$ [34].

To apply them, the classification results obtained for each class must be tagged individually as “True Positive” (*TP_c_*: belonging to a class and classified as the same class), “True Negative” (*TN_c_*: belonging to another class and classified as that class), “False Positive” (*FP_c_*: belonging to another class and classified to the evaluated class), or “False Negative” (*FN_c_*: belonging to the class and classified as other class). According to them, the high-level metrics are presented in the following equations:

(1) $$Accuracy=\sum_{c}\frac{TP_{c}+TN_{c}}{TP_{c}+FP_{c}+TN_{c}+FN_{c}},c\in classes$$

(2) $$Specificity=\sum_{c}\frac{TN_{c}}{TN_{c}+FP_{c}},c\in classes$$

(3) $$Precision=\sum_{c}\frac{TP_{c}}{TP_{c}+FP_{c}},c\in classes$$

(4) $$Sensitivity=\sum_{c}\frac{TP_{c}}{TP_{c}+FN_{c}},c\in classes$$

(5) $$F1_{score}=2\times \frac{precision\times sensitivity}{precision+sensitivity}.$$

About those metrics:

- *Accuracy*: All samples are classified correctly compared to all samples (see Equation (1)).
- *Specificity*: proportion of “true negative” values in all cases that do not belong to this class (see Equation (2)).
- *Precision*: Proportion of “true positive” values in all cases that have been classified as it (see Equation (3)).
- *Sensitivity* (or Recall): Proportion of “true positive” values in all cases that belong to this class (see Equation (4)).
- *F1${}_{score}$*: It considers both the precision and the sensitivity (recall) of the test to compute the score. It is the harmonic mean of both parameters (see Equation (5)).

There are other commonly used metrics, but not all works use them. However, the ROC curve (Receiver Operating Characteristic) [35] is of particular interest in diagnostic systems, because it is the visual representation of the True Positives Rate (TPR) versus the False Positives Rate (FPR) as the discrimination threshold is varied. Usually, when using the ROC curve, the area under the curve (AUC) is used as a value of the system’s goodness-of-fit.

### 3.4. Explainable AI

As mentioned in the introduction, it is, therefore, essential that the health professional has the possibility of accessing the justifications that have led the classifier to give a certain result. To this end, some tools have been developed in order to access various aspects related to the network’s decision-making.

Among all of them, the use of the Grad-CAM algorithm for CNN-based systems is very widespread [36]. The Gradient-weighted Class Activation Mapping (Grad-CAM) uses the gradients of any target concept, flowing into the final convolutional layer, to produce a coarse localization map highlighting the important regions in the image for predicting the concept.

Unlike the usual application of this algorithm, in this work, we apply it to an ensemble network. To do so, it is necessary to combine the Grad-CAM results from each of the networks that form the ensemble. To date, we are not aware of any other work that has used the Grad-CAM algorithm on an ensemble network formed by network models pre-trained with transfer learning techniques. In Section 4, the results obtained are detailed.

## 4. Results and Discussion

In this section, results may be presented in depth, and those results will be discussed as they are presented. First, the classification results are detailed and, after that, the explainable AI system results are presented.

### 4.1. Classification Results

This section presents the results obtained after the training process for each model or ensemble. These results are shown by the most commonly used metrics (described earlier) and the confusion matrixes. First, the individual model results are detailed; and then the ensemble results are shown.

#### 4.1.1. Individual CNN Results

In this subsection, the results for each individual model are detailed. These models are VGG-16, VGG-19, ResNet50, MobileNet-V2 and EfficientNet-B0.

##### VGG-16

The first individual model evaluated is VGG-16. The results obtained for this model are detailed in Table 3. As can be observed, global accuracy exceeds 91% on average, obtaining a value of almost 97% for the “monkeypox” class.

**Figure 3:**
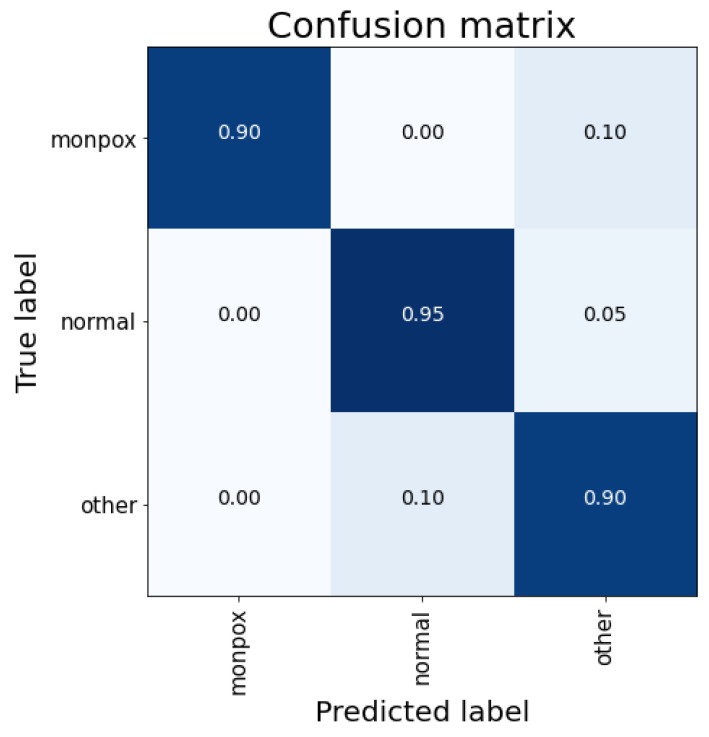
Confusion matrix for VGG-16 classification. Note: the higher the percentage of samples placed in a box, the higher the intensity of the blue colour.

Figure 3 shows the classification details for the test subset. As shown in the confusion matrix, there are no false positives for the “monkeypox” class. However, 10% failures of Monkeypox samples are classified as other skin damages; therefore, there are false negatives, which are the most dangerous cases.

##### VGG-19

The second model is VGG-19. The results obtained for this model are detailed in Table 4. As can be observed, global accuracy exceeds 93% on average, obtaining a value of almost 97% for the monkeypox class. These results are better than those obtained for the VGG-16 model; although, individually, the only class that improves its own accuracy is the “other skin damages” class.

**Figure 4:**
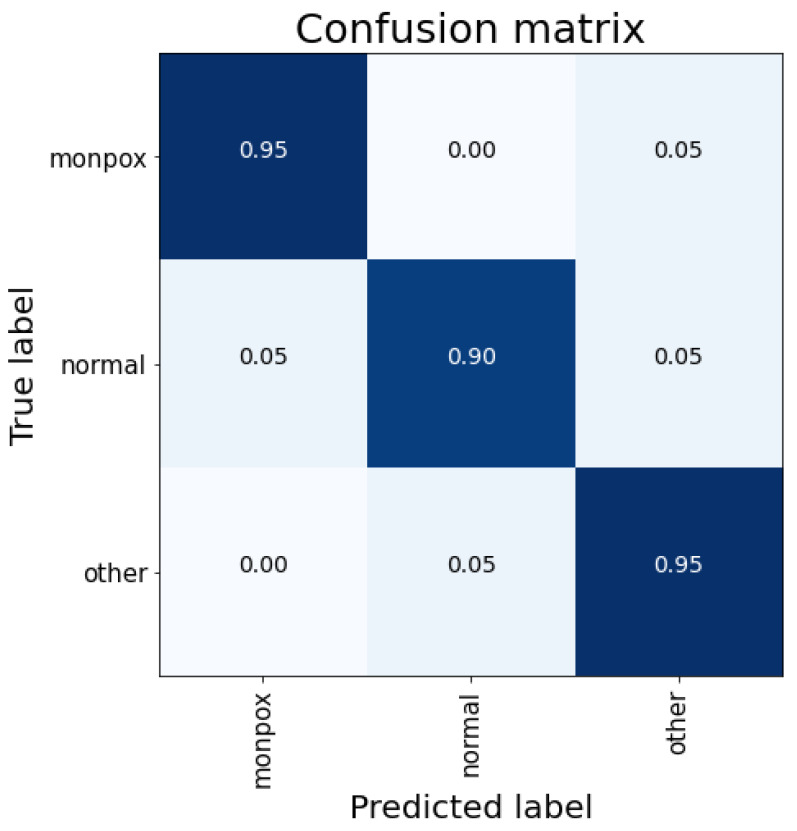
Confusion matrix for VGG-19 classification. Note: the higher the percentage of samples placed in a box, the higher the intensity of the blue colour.

These results can be easily observed in Figure 4. For this case, there are false positive cases for the “monkeypox” class (5% of the “healthy” class), although false negative cases are reduced to 5% (classifying them as “other skin damages”).

##### ResNet50

The next model is ResNet50. The results obtained for this model are detailed in Table 5. As can be observed, the global accuracy is 95% on average, obtaining a value of almost 97% for each class individually. These results are the best obtained so far (better than those obtained for the VGG-19 model).

**Figure 5:**
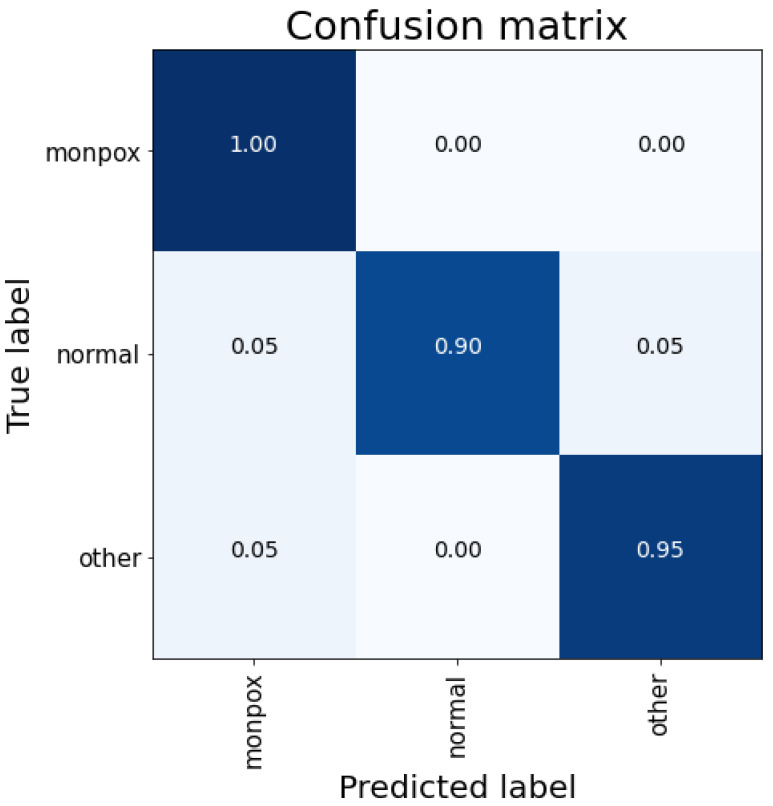
Confusion matrix for ResNet50 classification. Note: the higher the percentage of samples placed in a box, the higher the intensity of the blue colour.

Moreover, there is an essential difference between the ResNet50 and VGG-19 results, as can be seen in Figure 5. This difference relies on the absence of false negatives for the monkeypox class. For this case, the false negative decreasing is changed for an increase of false positive cases. Both the accuracy and the absence of false negatives make this model the most efficient for diagnostic purposes.

##### MobileNet-V2

Next, the MobileNet-V2 model is evaluated. The results obtained for this model are detailed in Table 6. For this case, the global accuracy decreases below 89%, being the worst evaluated model to date. Not surprisingly, there is a substantial drop in the accuracy of both the “monkeypox” class and the “other skin damages” class.

**Figure 6:**
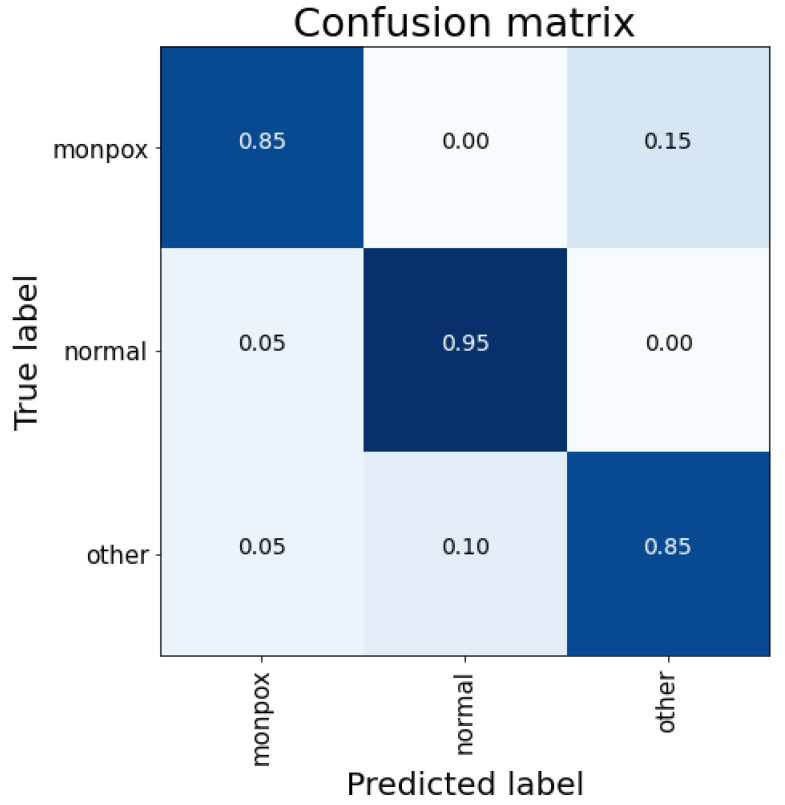
Confusion matrix for MobileNet-V2 classification. Note: the higher the percentage of samples placed in a box, the higher the intensity of the blue colour.

These data can be verified by looking at the confusion matrix in Figure 6. For the monkeypox class, 15% of the samples are classified as “other skin damages” (false negatives) and 5% of the samples of the other two classes are classified as monkeypox (false positives). As detailed above, this model obtains the worst results, mainly due to those false negatives (detailed by the low value of sensitivity).

##### EfficientNet-B0

The last individual model evaluated is EfficientNet-B0. The results obtained for this model are detailed in Table 7. The results are now better than those obtained with the MobileNet-V2 model (global accuracy of 90% and monkeypox accuracy of almost 97%), but these results are worse than those obtained with the ResNet50 model.

**Figure 7:**
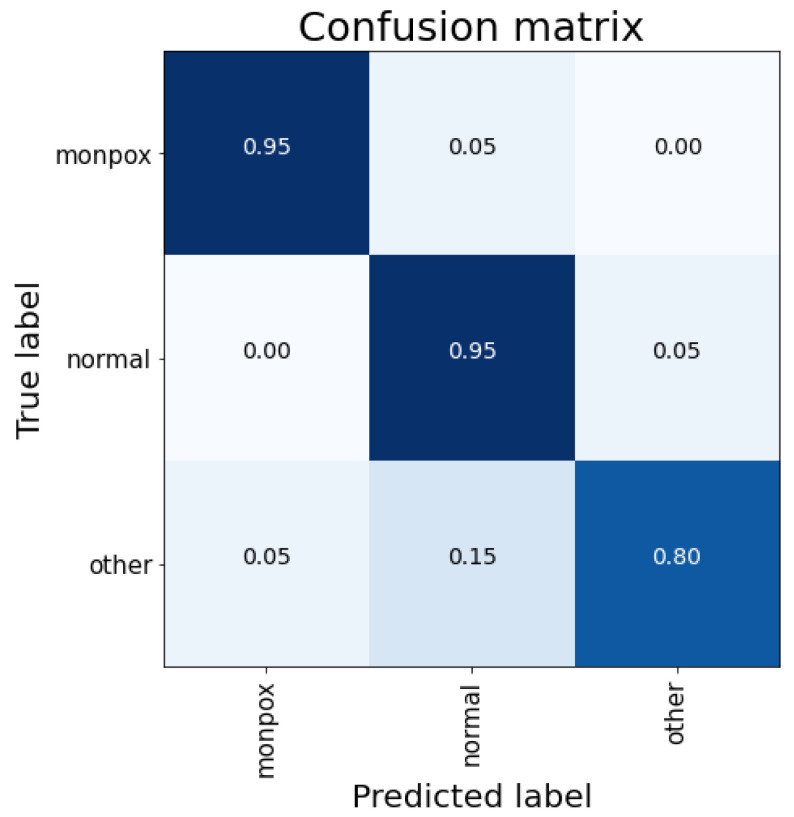
Confusion matrix for EfficientNet-B0 classification. Note: the higher the percentage of samples placed in a box, the higher the intensity of the blue colour.

As can be observed in Figure 7, both false negatives and false positives are reduced to 5% for each, but they are not completely eliminated. So, although these results are better than those obtained with the MobileNet-V2 model, they are worse than those obtained with the rest of the evaluated models.

Therefore, the best classification results obtained so far are those achieved with the ResNet50 model. Next, the three different ensemble networks are evaluated.

#### 4.1.2. Ensemble CNN Results

As indicated previously, the three ensemble classifiers evaluated will consist of: *[VGG-16 + VGG-19 + ResNet50]*, *[VGG-16 + ResNet50 + EfficientNet-B0]* and *[ResNet50 + EfficientNet-B0 + MobileNet-V2]*. The results of these ensembles will be combined by concatenation, simple average, and weighted average. Therefore, in addition to the ensembles, the mechanism used to combine the results will be evaluated.

There are multiple combinations of the networks, and in preliminary work, we have evaluated up to 6 combinations of 3 in 3 networks among those presented individually. The three ensemble networks presented in this paper are the ones that have obtained an accuracy higher than 90%.

##### VGG16 + VGG19 + ResNet50

For this first ensemble, the results obtained for each combination mechanism are detailed in Table 8 (concatenation), Table 9 (simple average) and Table 10 (weighted average).

**Figure 8:**
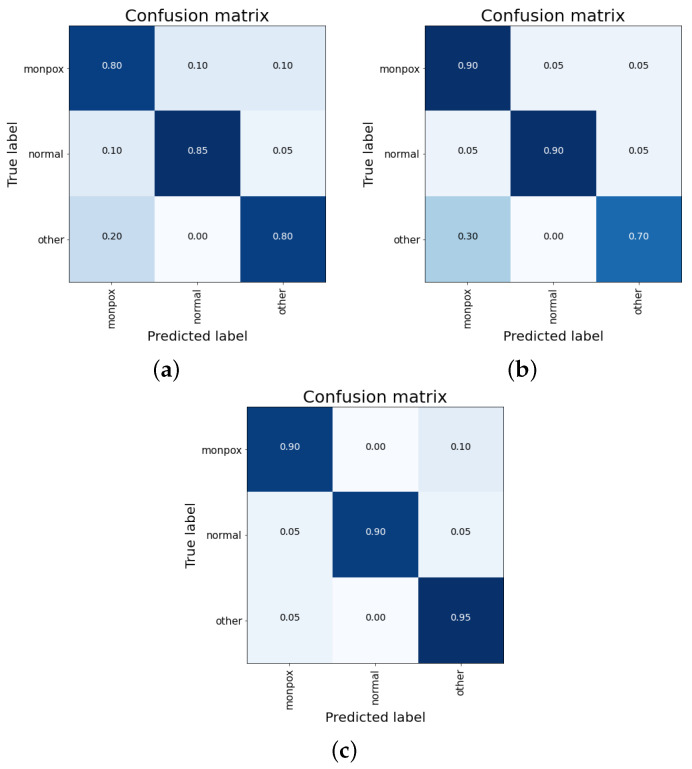
Confusion matrix for the ensemble composed by VGG-16, VGG-19 and ResNet50. Mixed results obtained by (**a**) concatenation, (**b**) simple average and (**c**) weighted average. Note: the higher the percentage of samples placed in a box, the higher the intensity of the blue colour.

The best results for this ensemble are obtained using the weighted average mechanism (see Table 10). Here, a global accuracy of almost 92% is obtained, very similar to the best result obtained with individual models (93.33% with ResNet50); however, the increase in computational cost does not justify the use of this ensemble. The main problems of this ensemble are the false negatives for the monkeypox class (10% of monkeypox samples), as can be observed in Figure 8.

##### VGG16 + ResNet50 + EfficientNetB0

For the second ensemble, the results obtained for each combination mechanism are detailed in Table 11 (concatenation), Table 12 (simple average), and Table 13 (weighted average).

As happened for the previous ensemble, the best results are obtained with the weighted average and, moreover, the accuracy results are the same too. Moreover, both the individual accuracy of each class and the false positives and negatives are the same. Therefore, the problem is the same as before: this system is too computationally complex for the results obtained (especially when comparing them with the individual ResNet50 model).

To date, the ensembles developed for this work do not obtain better accuracy results than those obtained with individual models. However, the last ensemble is analysed in the next subsection.

##### ResNet50 + EfficientNet + MobileNetV2

For this last ensemble network, the results are detailed in the same way as for the previous ones, dividing them by the combination mechanism: concatenation (see Table 14), simple average (see Table 15), and weighted average (see Table 16).

**Figure 9:**
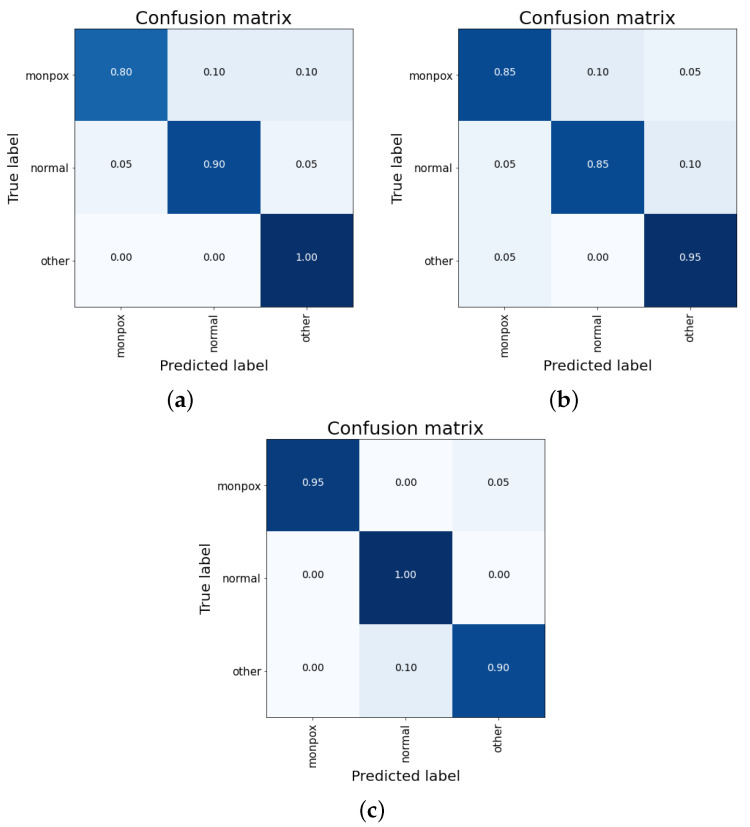
Confusion matrix for the ensemble composed by VGG-16, ResNet50 and EfficientNet-B0. Mixed results obtained by (**a**) concatenation, (**b**) simple average and (**c**) weighted average. Note: the higher the percentage of samples placed in a box, the higher the intensity of the blue colour.

For this last ensemble, the results obtained are better than all the previous ones. For combinations of simple average and weighted average, an overall accuracy greater than 98% is obtained. Moreover, for the “healthy” class, the accuracy is 100%, while for the other two classes (including “monkeypox”), the accuracy obtained is 98.33% (Figure 9).

**Figure 10:**
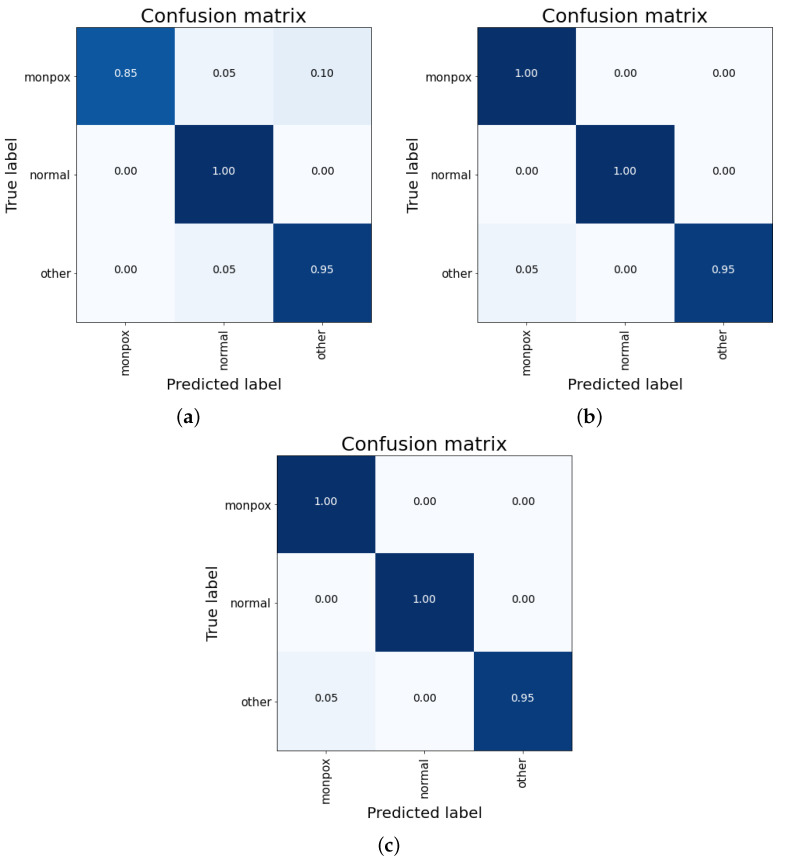
Confusion matrix for the ensemble composed by ResNet50, EfficientNet-B0 and MobileNet-V2. Mixed results obtained by (**a**) concatenation, (**b**) simple average and (**c**) weighted average. Note: the higher the percentage of samples placed in a box, the higher the intensity of the blue colour.

As can be seen in the confusion matrixes (see Figure 10), for the cases of a simple and weighted average (Figure 10a,b), no false negatives are observed (sensitivity value of 100%), although there is a 5% false positives cases from the “other skin damages” class.

Summarizing the results, The results obtained with this ensemble are almost a 4% better than those obtained so far with the individual ResNet50 model. The final results obtained for the best classifier include no misclassifications for both “monkeypox” and “healthy” classes, and a 5% misclassified samples for the “other skin damages” class (that are classified as “monkeypox”).

Table 17 summarizes the accuracy results obtained. In this table, columns 2 to 4 show the results obtained depending on the mechanism used by the ensemble to combine the results; this is the reason why these columns are not filled for individual models.

Looking at the information presented in Table 17, the best results are those obtained with the individual model ResNet50, and with the ensemble formed by ResNet50 + EfficientNet-B0 and MobileNet-V2. Of all these cases, the individual model obtained an accuracy of 95% and the ensemble an accuracy of 98.33%, improving the results of the individual models by 3.33%.

It could be considered whether it is worth using an ensemble network consisting of three individual networks to improve by more than a 3%; however, it is important to note that, analyzing each class individually, an improvement is obtained in all of them: 2% improvement in the “monkeypox” class (keeping false negatives to zero and reducing false positives); 4% improvement for the “healthy” class (reducing both false positives and false negatives to zero); and 2% improvement in the “other skin damages” class (reducing false positives to zero, while slightly maintaining some false negatives). In any case, all the indicators analyzed show an improvement using the ensemble network.

### 4.2. Explainable AI Results

This section presents the results obtained from the application of the Grad-CAM algorithm to the best-performing ensemble of those previously analyzed. That is, the one formed by the following three pre-trained models: ResNet50, EfficientNet-B0, and MobileNet-V2.

The implemented Explainable Deep Learning algorithm (custom Grad-CAM) extracts the resulting information after the last convolution layer (numerical weight matrix) and converts it to a heat map for each of the pre-trained models included in the ensemble. The results obtained from them are combined using the same algorithm as the ensemble itself. The result is the full ensemble heat map that shows the areas on which the classifier has been focused to obtain the verdict. This heat map is overlapped with the original image so that the health professional can appreciate the areas that determine the verdict.

However, if only the overlapped image is presented to the healthcare professional, he/she may not be able to see the affected area properly. This is why, in the final report, the original image and a text report with the percentage of confidence of belonging to each class are also provided.

**Figure 11:**
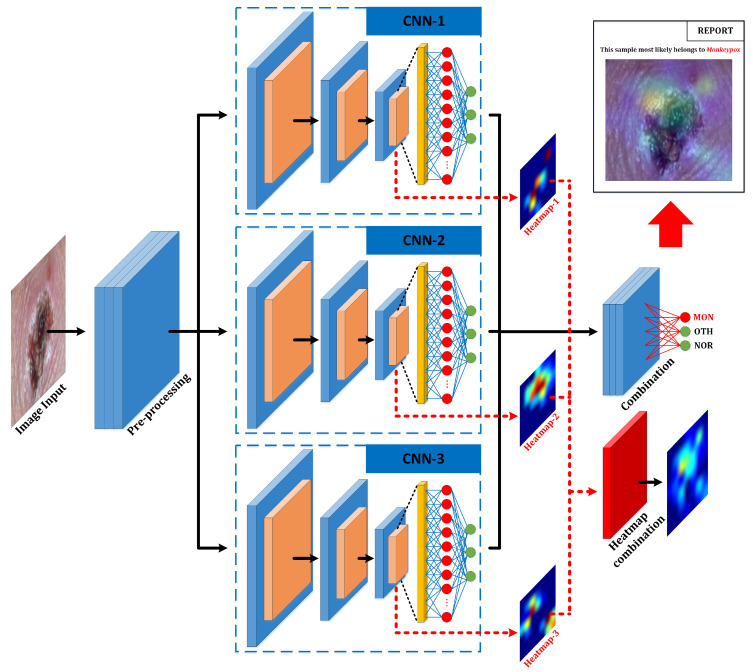
System’s final report given to the healthcare professional. Note: the arrows with continuous lines represent the flow of the result provided by the classifier, while the arrows with non-continuous lines represent the additional information extracted with the explainable AI technique and the additional results of the final report.

Figure 11 shows the original image with the overlapped heat map and the classification obtained provided. Based on these parameters, the healthcare professional can make the final verdict, which could be to validate these results or to proceed with a more thorough study of the sample.

**Figure 12:**
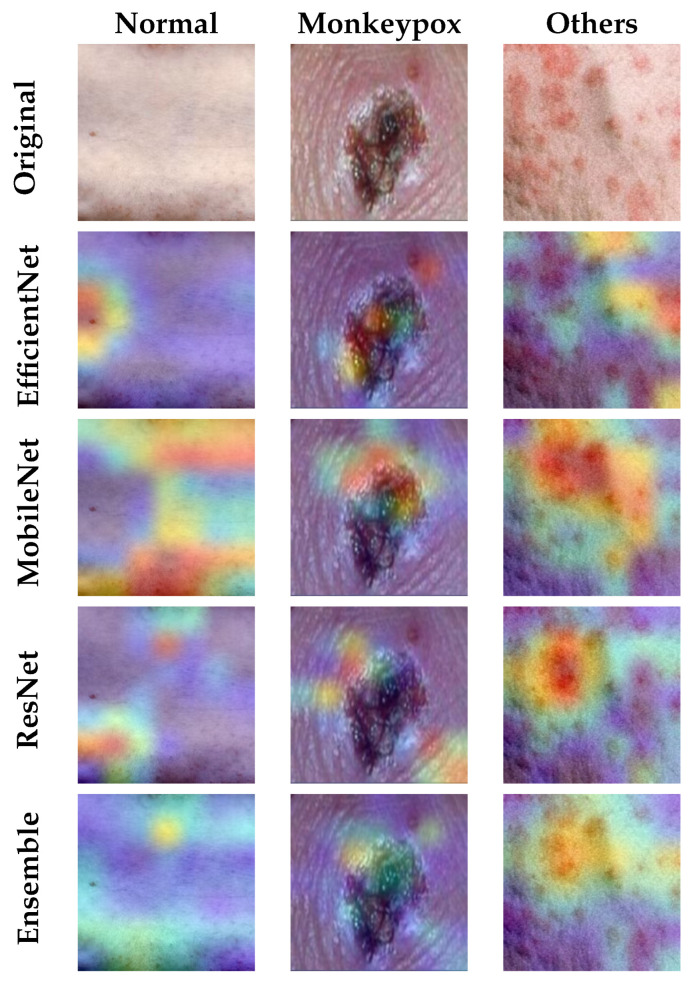
Results obtained for the Grad-CAM algorithm applied to each pre-trained model and to the ensemble.

Some reporting results for the training dataset are shown in Figure 12 regarding the Grad-CAM results for each pre-trained model individually and its comparison with the custom Grad-CAM of the ensemble.

Looking at the results shown for a specific example in Figure 11, several aspects can be appreciated:

- For the case of Monkeypox images, most systems focus individually on the central pustule of the image. However, it is true that in some cases they focus on the skin wrinkles produced by it. This situation, thanks to the Grad-CAM applied to the ensemble network, is solved by focusing on those aspects common to all networks. Not surprisingly, it can be seen for the ensemble that the resulting heatmap is located entirely on the pustule.
- For the normal skin case, discrepancies are observed between all the models individually, causing the ensemble heatmap to be more dispersed.
- Finally, for the case of other skin damage, being an example in which multiple spots and pimples appear, each model focuses its attention on a particular part of the image, although they all agree to focus on groups of skin spots and/or pimples. Coincidentally, the model with the best individual results (ResNet) focuses its attention on the area with the highest concentration of bumps. As for the ensemble heatmap, it also focuses on this group of bumps.

Summarizing, it can be observed that the result of applying the customized Grad-CAM on the ensemble network by combining the heatmaps of the pre-trained models involved, causes the attention on specific areas of the image to be diluted in favour of those areas common among all the models. Thus, if a model focuses its attention on an area on which the others are not focused, the ensemble will not take it into account in the final result.

### 4.3. Comparison with Previous Works

Finally, the results of the classifier developed in this work can be analysed in comparison with the two previous works detailed in Section 2. The comparative results are presented in Table 18.

If we look at the results obtained in previous work, the classifier developed in this work improves the overall accuracy of the system (in addition to the individual accuracy of all classes).

The first compared work uses its own two-class dataset and, therefore, its classifier is a two-class classifier (“Monkeypox” and “others”). The best classification results are obtained with the individual ResNet50 model (almost 83%). In this aspect, it is similar to our work, since the best results for individual models are obtained with the ResNet50 model; even so, our work improves the classification results for the ResNet50 model by a 12% (it should also be taken into account that our work uses three classes, not two). Finally, this first previous work also evaluates an ensemble (consisting of VGG-16 + ResNet50 + Inception-V3), obtaining worse results than those obtained with ResNet50.

The second compared work uses its own dataset too, but in this case it contains four classes. However, in that work, two two-class classifiers are developed: one classifies between “monkeypox” and “chickenpox”, and the other classifies between “monkeypox” and “others”. This work only analyses the VGG-16 model, obtaining classification results of 83% for the first case and 78% for the second one (taking into account the results presented for the test subset). If we compare our work with this one, we obtain an improvement of 9% using the VGG-16 model (although it is one of the worst results obtained in our work). In general, the improvement we obtain with respect to this work is more than 15%.

## 5. Conclusions

This works presents the need to develop a diagnostic aid for low-resolution images obtained with a mobile phone. To this end, the need for artificial intelligence techniques is justified as an initial screening mechanism to avoid saturation of emergency departments and also for areas where advanced diagnostic equipment is not available.

To this end, images have been collected from government sources to build a proprietary (and public) dataset that distinguishes between images of healthy skin, Monkeypox, and other skin diseases.

With this dataset, a process of classifier development and evaluation has been followed in which, in the first instance, several pre-trained models are trained with variations in the hyperparameters. In addition, combinations of these models are used to train different ensembles.

The results obtained exceed 98% accuracy for the best classifier.

With these results, we proceeded to compare this work with previous work on Monkeypox classification. The comparison shows an improvement in accuracy with respect to these other works.

In addition to the classification results, two added values are provided: the new dataset developed specifically for this work (which has more images and is better balanced than the existing ones), and the inclusion of explainable AI tools (which provide a more detailed report to the healthcare professional).

Ultimately, the three notable aspects of this work are the improvement of the classifier developed, the public dataset, and the application of xAI techniques.

## Figures and Tables

**Table 1:** Public monkeypox datasets compared with the dataset collected for this work. Clarification: the number inside the brackets indicate the number of classes labelled in the dataset.

Dataset	Classes	Number of Images	Type of Images
Ali (2022) [24]	2: Monkeypox, Others	102, 126	Full body, Limbs, Face, Trunk
Ahsan (2022) [25,26]	4: Healthy, Monkeypox, Chickenpox, Measles	54, 43, 47, 17	Full body, Limbs, Face, Trunk
MonkeypoxSkin dataset (this work) [27]	3: Healthy, Monkeypox, Other skin diseases	100, 100, 100	Close skin tissue

**Table 2:** Dataset elaborated for this work and subsets division.

Class	Original Dataset			Augmented Dataset		
	Train	Validation	Test	Train	Validation	Test
Healthy	60	20	20	3000	20	20
Monkeypox	60	20	20	3000	20	20
Other skin damages	60	20	20	3000	20	20
TOTAL	180	60	60	9000	60	60

**Table 3:** Classification results obtained for VGG-16 classifier.

Class	Accuracy	Specificity	Precision	Sensitivity	F1${}_{\mathrm{score}}$
Monkeypox	96.67	100	100	90	94.73
Healthy	95	95	90.47	95	92.68
Other skin damages	91.67	92.5	85.71	90	87.8
Global	91.67	95.83	91.67	91.67	91.67

**Table 4:** Classification results obtained for VGG-19 classifier.

Class	Accuracy	Specificity	Precision	Sensitivity	F1${}_{\mathrm{score}}$
Monkeypox	96.67	97.5	95	95	95
Healthy	95	97.5	94.73	90	92.31
Other skin damages	95	95	90.47	95	92.68
Global	93.33	96.67	93.33	93.33	93.33

**Table 5:** Classification results obtained for ResNet50 classifier.

Class	Accuracy	Specificity	Precision	Sensitivity	F1${}_{\mathrm{score}}$
Monkeypox	96.67	95	90.91	100	95.24
Healthy	96.67	100	100	90	94.74
Other skin damages	96.67	97.5	95	95	95
Global	95	97.75	95	95	95

**Table 6:** Classification results obtained for MobileNet-V2 classifier.

Class	Accuracy	Specificity	Precision	Sensitivity	F1${}_{\mathrm{score}}$
Monkeypox	91.67	95	89.47	85	87.18
Healthy	95	95	90.48	95	92.68
Other skin damages	90	92.5	85	85	85
Global	88.33	94.17	88.33	88.33	88.33

**Table 7:** Classification results obtained for EfficientNet-B0 classifier.

Class	Accuracy	Specificity	Precision	Sensitivity	F1${}_{\mathrm{score}}$
Monkeypox	96.67	97.5	95	95	95
Healthy	91.67	90	82.61	95	88.37
Other skin damages	91.67	97.5	94.12	80	86.49
Global	90	95	90	90	90

**Table 8:** Classification results obtained for the ensemble VGG-16 + VGG-19 + ResNet50. Combination results obtained by concatenation.

Class	Accuracy	Specificity	Precision	Sensitivity	F1${}_{\mathrm{score}}$
Monkeypox	83.33	85	72.72	80	76.19
Healthy	91.67	95	89.47	85	87.18
Other skin damages	88.33	92.5	84.21	80	82.05
Global	81.67	90.83	81.67	81.67	81.67

**Table 9:** Classification results obtained for the ensemble VGG-16 + VGG-19 + ResNet50. Combination results obtained by simple average.

Class	Accuracy	Specificity	Precision	Sensitivity	F1${}_{\mathrm{score}}$
Monkeypox	85	82.5	72	90	80
Healthy	95	97.5	94.74	90	92.31
Other skin damages	86.67	95	87.5	70	77.78
Global	83.3	91.67	83.33	83.33	83.33

**Table 10:** Classification results obtained for the ensemble VGG-16 + VGG-19 + ResNet50. Combination results obtained by weighted average.

Class	Accuracy	Specificity	Precision	Sensitivity	F1${}_{\mathrm{score}}$
Monkeypox	93.33	95	90	90	90
Healthy	96.67	100	100	90	94.74
Other skin damages	93.33	92.5	86.36	95	90.48
Global	91.67	95.83	91.67	91.67	91.67

**Table 11:** Classification results obtained for the ensemble VGG-16 + ResNet50 + EfficientNet-B0. Combination results obtained by concatenation.

Class	Accuracy	Specificity	Precision	Sensitivity	F1${}_{\mathrm{score}}$
Monkeypox	91.67	97.5	94.12	80	86.49
Healthy	93.33	95	90	90	90
Other skin damages	95	92.5	89.96	100	93.02
Global	90	95	90	90	90

**Table 12:** Classification results obtained for the ensemble VGG-16 + ResNet50 + EfficientNet-B0. Combination results obtained by simple average.

Class	Accuracy	Specificity	Precision	Sensitivity	F1${}_{\mathrm{score}}$
Monkeypox	91.67	95	89.47	85	87.18
Healthy	91.67	95	89.47	85	87.18
Other skin damages	93.33	92.5	86.36	95	90.48
Global	88.33	94.17	88.33	88.33	88.33

**Table 13:** Classification results obtained for the ensemble VGG-16 + ResNet50 + EfficientNet-B0. Combination results obtained by weighted average.

Class	Accuracy	Specificity	Precision	Sensitivity	F1${}_{\mathrm{score}}$
Monkeypox	93.33	95	90	90	90
Healthy	96.67	100	100	90	94.74
Other skin damages	93.33	92.5	86.36	95	90.48
Global	91.67	95.83	91.67	91.67	91.67

**Table 14:** Classification results obtained for the ensemble ResNet50 + EfficientNet-B0 + MobileNet-V2. Combination results obtained by concatenation.

Class	Accuracy	Specificity	Precision	Sensitivity	F1${}_{\mathrm{score}}$
Monkeypox	95	100	100	85	91.89
Healthy	96.67	95	90.91	100	95.24
Other skin damages	95	95	90.48	95	92.68
Global	93.33	96.67	93.33	93.33	93.33

**Table 15:** Classification results obtained for the ensemble ResNet50 + EfficientNet-B0 + MobileNet-V2. Combination results obtained by simple average.

Class	Accuracy	Specificity	Precision	Sensitivity	F1${}_{\mathrm{score}}$
Monkeypox	98.33	97.5	95.24	100	97.56
Healthy	100	100	100	100	100
Other skin damages	98.33	100	100	95	97.44
Global	98.33	99.17	98.33	98.33	98.33

**Table 16:** Classification results obtained for the ensemble ResNet50 + EfficientNet-B0 + MobileNet-V2. Combination results obtained by weighted average.

Class	Accuracy	Specificity	Precision	Sensitivity	F1${}_{\mathrm{score}}$
Monkeypox	98.33	97.5	95.24	100	97.56
Healthy	100	100	100	100	100
Other skin damages	98.33	100	100	95	97.44
Global	98.33	99.17	98.33	98.33	98.33

**Table 17:** Summarized results for all the classifiers.

Classifier	Accuracy Results			
	Concatenated	Simple Avr	Weighted Avr	Best
VGG-16	−	−	−	91.67
VGG-19	−	−	−	93.33
ResNet50	−	−	−	95
EfficientNet-B0	−	−	−	90
MobileNet-V2	−	−	−	88.33
VGG16 + VGG19 + ResNet	81.67	83.3	91.67	91.67
VGG16 + ResNet + EfficientNet	90	88.33	91.67	91.67
ResNet + EfficientNet + MobileNet	93.33	98.33	98.33	98.33

**Table 18:** Comparison with previous works.

Work	Dataset	Classes	Classifier	Results (%)
**Ali et al. [24]**	Own	2: Monkeypox, Others	VGG-16 ResNet50 Inception-V3 Ensemble	[VGG16] Acc: 81.48, Pre: 85, Sen: 81, F1: 83 [ResNet] Acc: 82.96, Pre: 87, Sen: 83, F1: 84 [Inception] Acc: 74.03, Pre: 74, Sen: 81, F1: 78 [Ensemble] Acc: 79.26, Pre: 84, Sen: 79, F1: 81
**Ahsan et al. [25]**	Own	2: Monkeypox, Chickenpox 2: Monkeypox, Others	VGG-16	[Case 1] Acc: 83, Pre: 88, Sen: 83, Spe: 66, F1: 83 [Case 2] Acc: 78, Pre: 75, Sen: 75, Spe: 83, F1: 75
**This work (2022)**	MonkeypoxSkin	3: Healthy, Monkeypox, Other skin diseases	VGG-16 VGG-19 ResNet50 MobileNet-V2 EfficientNet-B0 Ensemble 1 Ensemble 2 Ensemble 3	[VGG16] Acc: 91.67, Pre: 92, Sen: 92, Spe: 96, F1: 92 [VGG19] Acc: 93.33, Pre: 93, Sen: 93, Spe: 97, F1: 93 [ResNet] Acc: 95, Pre: 95, Sen: 95, Spe: 97.75, F1: 95 [MobileNet] Acc: 88.33, Pre: 88, Sen: 88, Spe: 94, F1: 88 [EfficientNet] Acc: 90, Pre: 90, Sen: 90, Spe: 95, F1: 90 [Ensemble1] Acc: 91.67, Pre: 92, Sen: 92, Spe: 96, F1: 92 [Ensemble2] Acc: 91.67, Pre: 92, Sen: 92, Spe: 96, F1: 92 [Ensemble3] Acc: 98.33, Pre: 98, Sen: 98, Spe: 99, F1: 98

## Data Availability

Public available dataset in https://github.com/mjdominguez/MonkeypoxSkinImages, accessed on 15 May 2023.
